# Formamidinium Lead Bromide (FAPbBr_3_) Perovskite Microcrystals for Sensitive and Fast Photodetectors

**DOI:** 10.1007/s40820-018-0196-2

**Published:** 2018-04-02

**Authors:** Fengying Zhang, Bin Yang, Kaibo Zheng, Songqiu Yang, Yajuan Li, Weiqiao Deng, Rongxing He

**Affiliations:** 1 Key Laboratory of Luminescence and Real-Time Analytical Chemistry of Ministry of Education, College of Chemistry and Chemical Engineering, Southwest University, Chongqing, 400715 People’s Republic of China; 2 State Key Laboratory of Molecular Reaction Dynamics, Dalian Institute of Chemical Physics, Chinese Academy of Science, Dalian, 116023 People’s Republic of China; 3 Department of Chemical Physics and NanoLund Chemical Center, Lund University, P.O. Box 124, 22100 Lund, Sweden

**Keywords:** Formamidinium lead trihalide (FAPbBr_3_), Perovskite microcrystals, Photodetector, External quantum efficiency

## Abstract

Because of the good thermal stability and superior carrier transport characteristics of formamidinium lead trihalide perovskite HC(NH_2_)_2_PbX_3_ (FAPbX_3_), it has been considered to be a better optoelectronic material than conventional CH_3_NH_3_PbX_3_ (MAPbX_3_). Herein, we fabricated a FAPbBr_3_ microcrystal-based photodetector that exhibited a good responsivity of 4000 A W^−1^ and external quantum efficiency up to 10^6^% under one-photon excitation, corresponding to the detectivity greater than 10^14^ Jones. The responsivity is two orders of magnitude higher than that of previously reported formamidinium perovskite photodetectors. Furthermore, the FAPbBr_3_ photodetector’s responsivity to two-photon absorption with an 800-nm excitation source can reach 0.07 A W^−1^, which is four orders of magnitude higher than that of its MAPbBr_3_ counterparts. The response time of this photodetector is less than 1 ms. This study provides solid evidence that FAPbBr_3_ can be an excellent candidate for highly sensitive and fast photodetectors.

**Electronic supplementary material:**

The online version of this article (10.1007/s40820-018-0196-2) contains supplementary material, which is available to authorized users.

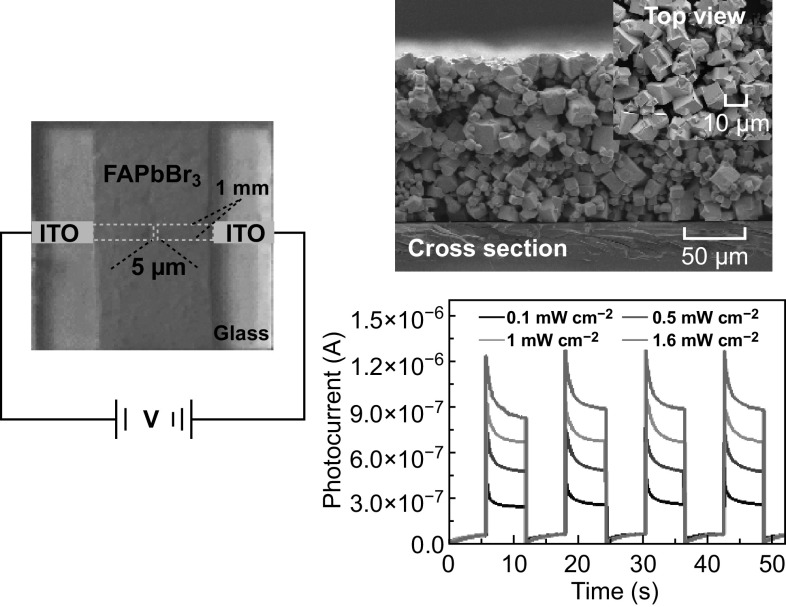

## Highlights

- Formamidinium lead trihalide (FAPbBr_3_) microcrystal-based photodetectors facilitate efficient charge transfer.
- The fabricated FAPbBr_3_ photodetector shows good responsivity, external quantum efficiency, and detectivity.
- Two-photon performance of the photodetectors is better than that previously reported for MAPbBr_3_ photodetectors.

## Introduction

In recent years, photoelectric devices have been widely investigated [1–3], especially detectors with different morphologies and sizes, for exploring their mechanisms, simplifying their syntheses, and improving device efficiencies [4–10]. Photodetectors based on hybrid organolead trihalide perovskite CH_3_NH_3_PbX_3_ (MAPbX_3_, X = Cl, Br, I) have attracted significant attention in the optoelectronic field owing to their strong absorption coefficients (up to 10^5^ cm^−1^) [11–14], carrier mobilities (2.5–1000 cm^2^ v^−1^ s^−1^) [15–19], long carrier lifetimes (0.08–4.5 μs) [17, 19–21], and diffusion lengths (2–175 μm) [19, 20, 22, 23]. In particular, Saidaminov et al. produced a planar-integrated photodetector using MAPbBr_3_ single crystals, achieving high-performance light detection in the wide wavelength range [24]. Bao et al. reported low-noise and large-linear-dynamic-range photodetectors based on MAPbBr_3_ and MAPbI_3_ [25]. Yang et al. demonstrated the great potential of metal–semiconductor–metal structures for low-cost and high-performance optoelectronic devices [26]. Moreover, Hu et al. [27] reported high-performance and low working-voltage perovskite thin-film photodetectors. Further, a UV-selective photodetector based on MAPbCl_3_ was also demonstrated [28, 29]. However, conventional MAPbX_3_ materials have poor stability [30, 31], which severely impedes device development.

HC(NH_2_)_2_PbX_3_ (FAPbX_3_), which replaces organic methylammonium cation (MA^+^) with the larger formamidinium cation (FA^+^) [32, 33], is much more stable in air because the high Goldschmidt tolerance factor of its lattice (≈ 1) [33–35]. Moreover, previous reports have revealed that FA-substituted compounds (e.g., FAPbX_3_) showed remarkably improved carrier transmission performance than MAPbX_3_ in both polycrystalline thin films and monocrystalline phases, including a longer carrier lifetime and lower dark carrier concentration [20, 22, 35–37]. As a result, FAPbBr_3_ is expected to perform better in photodetectors than MAPbBr_3_. However, a prototype device based on FAPbBr_3_ has not been reported yet, except for a two-dimensional (OA)_2_FA_*n*−1_Pb_*n*_Br_3*n*+1_ photodetector with low responsivity [38]. Taking advantage of the high carrier mobility, stability of single crystals and the efficient charge transfer in the micron scale, we synthesized FAPbBr_3_ microcrystals (MCs) and deposited them as a photodetector. Detailed investigations under single- and two-photon excitation and front- and back-side excitation were performed to reveal the great potential of the fabricated photodetector as an optoelectronic device.

## Experimental

### Chemicals and Reagents

Formamidine acetate, lead bromide (PbBr_2_, > 98%) and hydrobromic acid (48 wt% in water) were purchased from Alfa Aesar. Gamma-butyrolactone (GBL) and N,N-dimethylformamide (DMF) were purchased from Kermel. All compounds were used without any further purification.

### Synthesis of CH(NH_2_)_2_Br (FABr)

FABr was synthesized by slowly dropping 10 mL hydrobromic acid into 50 mmol (5.205 g) formamidine acetate in a flask, accompanied by continuous stirring at 0 °C for 2 h under argon atmosphere. The product FABr was formed once the solvent was removed using a rotary evaporator at 70 °C. The crude white powder was dissolved in ethanol and subsequently reprecipitated in diethyl ether. Then, the filtered product was dried at 60 °C in a vacuum oven for 24 h for further use.

### Synthesis of FAPbBr_3_ MCs

FAPbBr_3_ MCs were grown by a modified inverse temperature crystallization method using toluene as the antisolvent. In brief, 5 mmol PbBr_2_ (1.835 g) and 5 mmol FABr (0.625 g) were dissolved in 10 mL mixed DMF:GBL (1:1 v/v) solvent at 25 °C, and the solution was filtered using nylon filters with a 0.22-μm pore size. Then, 1 mL precursor was diluted in 2 mL DMF and antisolvent toluene was added to obtain a saturated solution. Single crystals of FAPbBr_3_ with millimeter dimensions were formed after stirring on a hot plate of 80 °C.

### Device Preparation

Standard photolithography and hydrochloric acid etching were used to obtain conductive glass substrates with a channel length and width of 5 µm and 1 mm, respectively. After synthesizing FAPbBr_3_ MCs using the above-described method, the substrates were deposited on these crystals and dried at 160 °C for 10 min.

### Measurements and Characterizations

Scanning electron microscopy (SEM) was performed using a field-emission scanning electron microscope (JEOL, JSM-7800F, 3 kV). X-ray diffraction (XRD) was performed using an X’pert PRO diffractometer equipped with Cu K_α_ X-ray (*λ* = 1.54186 Å) tubes. UV–Vis diffuse reflectance spectra were recorded at room temperature on a JASCO V-550 UV–Vis absorption spectrometer with an integrating sphere operating in 200–900 nm. Photoluminescence (PL) was recorded using a Horiba PTI QuantaMaster 400 system with an excitation of 470 nm. Time-resolved PL decay was obtained on the basis of the time-correlated single-photon counter (TCSPC) technology using a light-emitting diode (LED) to provide a 376-nm excitation beam. The decay data were analyzed using commercial software provided by Horiba. For trap-state density evaluation, one layer of conductive glass (ITO) undertaken the crystal film was used as one electrode. An 800-nm-thick gold (Au) layer deposited on top of the film by thermal evaporation was used as the other electrode. This structure had a rather simple geometry with the sample deposited on ITO and evaporated Au on opposite sides, and the structure should be kept in the dark. Current–voltage measurements were conducted using a Keithley 2400 source meter. For light characterization under one-photon excitation, a monochromatic source (LED, *λ* = 495 nm) was used. Spectral responsivity (*R*) was calculated by the photocurrent (*I*_ph_) and incident power (*P*_inc_) according to the relation *R *= *I*_ph_/*P*_inc_. For two-photon irradiation, the photocurrent was generated using a femtosecond laser system (Spitfire Pro, SpectraPhysic) with an output wavelength of 800 nm and a repetition rate of 1000 Hz as the light source.

## Results and Discussion

**Fig. 1:**
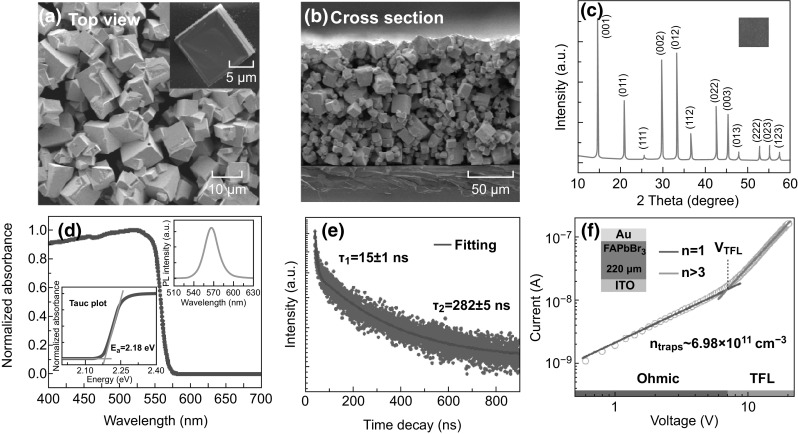
**a** Top-view and **b** cross-sectional SEM images. **c** XRD pattern **d** Steady-state absorption spectrum of FAPbBr_3_ MCs film. Left insets: the optical bandgap extracted from Tauc plot; right insets: PL spectrum. **e** PL decay of the sample excited at 376 nm, which could be well fitted by biexponential functions. **f** Current–voltage responses of FAPbBr_3_ MCs

We synthesized FAPbBr_3_ MCs using a modified antisolvent-assisted inverse temperature crystallization method [8, 24, 28]. To facilitate the removal of the solvent and form homogeneous crystalline films, 1 M precursor (equimolar FABr and PbBr_2_ dissolved in a mixed solvent as shown in experimental section) was diluted three times in DMF. The antisolvent toluene was then added to obtain a saturated solution. This saturated solution was stirred at 80 °C to accelerate the nucleation and increase the yield of the interconnected crystals. The mean size of the as-obtained FAPbBr_3_ MCs was ~ 10 ± 5 µm, as shown in the top-view SEM image (Fig. 1a). These MCs were interconnected as a continuous film with a thickness of ~ 150 µm, as shown in Fig. 1b. XRD results shown in Fig. 1c confirmed the cubic phase of the FAPbBr_3_ MCs [35, 39]. The MCs exhibited an absorption band edge at 570 nm, corresponding to a bandgap of 2.18 eV, as obtained from the Tauc plot of the absorption spectrum (Fig. 1d). The emission peak of the MCs appeared at 567 nm (insets of Fig. 1d**)**, which was consistent with previous results [33, 37, 40]. Additionally, PL decay was measured using the TCSPC technology (Fig. 1e), and a fast component (*τ*_1_, 15 ± 1 ns) and slow decay (*τ*_2_, 282 ± 5 ns) reflected the surface and bulk carrier lifetimes of the FAPbBr_3_ MCs, respectively. We also conducted dark current measurements to estimate the trap-state density of the FAPbBr_3_ MC film, as depicted in Fig. 1f. Here, the trap-state density was calculated to be 6.98 × 10^11^ cm^−3^ according to Eq.1:

1 $$n_{\text{trap}} = \frac{{2\varepsilon_{0} \varepsilon V_{\text{TFL}} }}{{qL^{2} }}$$

where *V*_TFL_ is the voltage at which trap states are fully filled by injected carriers, *q* and *L* represent the elemental charge and film thickness, respectively, *ɛ*_0_ and *ɛ* denote the vacuum permittivity and dielectric constant of FAPbBr_3_, with *ɛ* = 43.6 [37]. The relatively fewer defects contributed to the formation of a high-quality film, thus promoting their application in photoelectronic devices [37, 41].

**Fig. 2:**
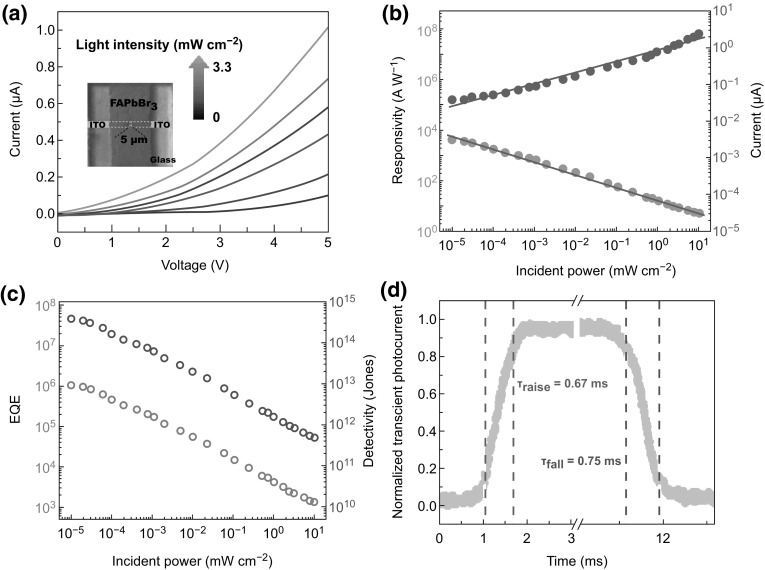
**a** Current–voltage characteristics measured under back-side excitation with varying light intensity. **b** Responsivity and corresponding current as a function of incident light power under back-side illumination. The detector was biased at 5 V. **c** EQE and detectivity under different light illuminations. **d** Response time measured by a monochromatic source of 495 nm under back-side illumination and rise time and fall time

In the next step, FAPbBr_3_ MCs were directly deposited on interdigitated ITO substrates to form the prototype photodetector device. The length and width of the gaps between neighboring digits were 5 µm and 1 mm, respectively, as shown in Fig. S1. FAPbBr_3_ MCs covered the entire active area, forming Schottky barriers due to contact with the ITO electrode. Once a voltage was applied to the detector device, ion migration and carrier trapping occurred in the active layer and at the electrode/perovskite interfaces, respectively. This indicated that an Ohmic contact between the FAPbBr_3_ MCs and the ITO electrode was formed. The cathode collected abundant photogenerated holes, while holes from the anode were injected into the active layer, showing the photoconductivity of the device [42–44]. The compact morphology of the MCs minimized the grain boundary and consequently diminished interfacial charge recombination. As shown in Fig. 2a, the photocurrent drastically increased with increasing excitation light intensity. In addition, the small dark current of the device (seen in Fig. S2) indicated the low carrier concentration of FAPbBr_3_ MCs [37]. The photoresponse was also reproducible under a periodic excitation of the light pulse, as demonstrated in Fig. S3.

As shown in Fig. 2b, the photocurrent increased linearly with the incident power and the corresponding responsivity (*R*, defined as photocurrent/the incident light power) linearly decreased. The responsivity was 4000 A W^−1^ at 5 V (*λ* = 495 nm, probe intensity = 10 nW cm^−2^), which was two orders of magnitude higher than that of other FA-based perovskite photodetectors [7, 38, 45, 46]. Furthermore, the external quantum efficiency (EQE) calculated by Eq. 2 was as high as 1.05 × 10^6^% (Fig. 2c):

2 $${\text{EQE}} = \frac{{R \cdot {\text{hc}}}}{q\lambda }$$

In addition, the detectivity D* of the device was 3.87 × 10^14^ Jones, as obtained from Eq. 3:

3 $$D^{*} = \frac{R\sqrt A }{{\sqrt {qI_{\text{dark}} } }}$$

This value of D* was higher than that of state-of-art MAPbI_3_ photodetectors (~ 10^13^ Jones) [46]. Moreover, the FAPbBr_3_ MC photodetector exhibited a rapid response with a rise time (*τ*_rise_) of 0.67 ms and fall time (*τ*_fall_) of 0.75 ms (Figs. 2d and S4**)**, where *τ*_rise_ and *τ*_fall_ are defined as the time required for light response from 10 to 90% in the rising stage and from 90 to 10% in the falling stage, respectively.

**Fig. 3:**
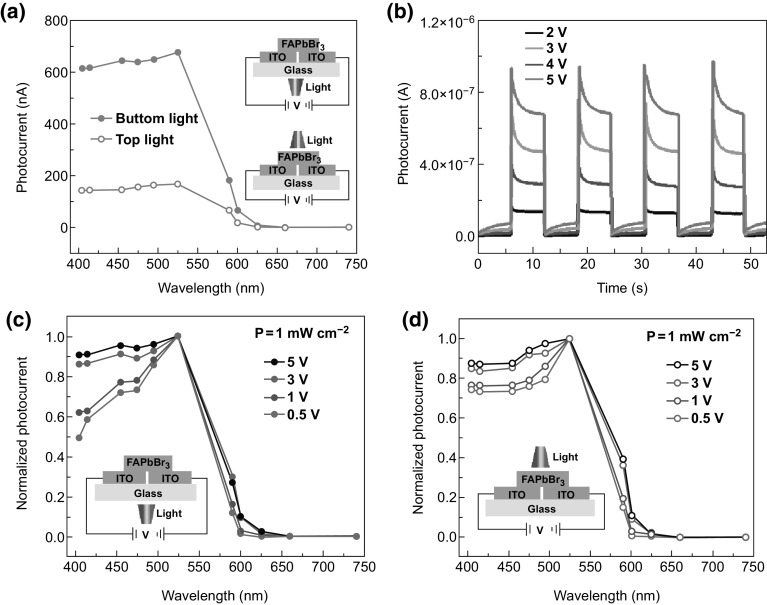
**a** Spectral-dependent photocurrent under front-side and back-side excitation at 1 mW cm^−2^ and a bias voltage of 5 V. **b** Photocurrent response measured using a series of bias voltages: 2, 3, 4, and 5 V, at an excitation of 495 nm. Normalized spectral-dependent photocurrent under different bias voltages under **c** back-side and **d** front-side excitation

Previous research has revealed that the photoresponse to the excitation wavelength is different for front-side excitation and back-side excitation [46]. In back-side excitation, charge carriers are efficiently collected in the vicinity of the electrodes. However, in front-side excitation, it is more difficult for charge carriers generated by short-wavelength photons to penetrate the thick film to electrodes than that of long-wavelength photons with energy comparable to the bandgap [46]. In our case, the wavelength-dependent light response using front-side excitation resembled that using back-side excitation, indicating that our device was a broadband photodetector (Figs. 3a and S5). It was found that the thickness of the FAPbBr_3_ microcrystalline film of about 150 µm allowed most photons to be transmitted through the film to generate corresponding photocurrents. Once the film became sufficiently thick (> 200 µm), it blocked the short-wavelength photons to the microcrystalline film to form a narrowband photodetector, which was also confirmed by Saidaminov and coworkers [46]. In addition, the generally lower photocurrent for front-side excitation could be attributed to the insufficient diffusion length of the photogenerated charges for the given film thickness, which hindered charge transportation to the bottom electrode [46]. Figure 3b shows the photocurrent variation for different bias voltages. As shown in Fig. 3c, d, when the bias voltage was decreased, the photocurrents of the photons excited far above the absorption band edge degraded more.

**Fig. 4:**
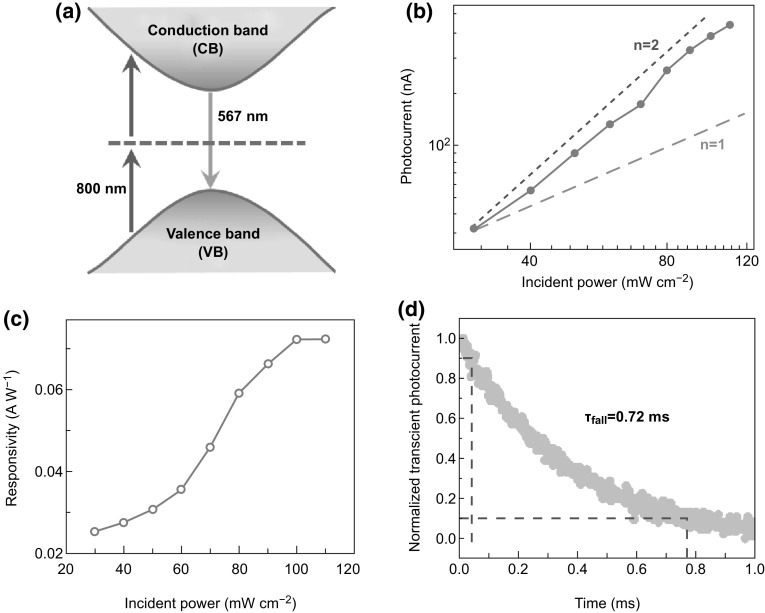
**a** Schematic showing two-photon absorption of 800 nm light and up-conversion to 567 nm PL. **b** Photocurrent and **c** responsivity dependence at an input light intensity of 800 nm and a bias of 5 V. **d** Fall time measured using pulsed light of 800 nm

Perovskites have also attracted considerable attention as nonlinear semiconductor absorbers for optical limiting [47], ultrafast optical signal characterization [48], microscopy [49], and lithography [50]. Therefore, we characterized our device under two-photon excitation, where the photocurrent was generated by an 800-nm pulse laser with a photon energy much smaller than the bandgap of FAPbBr_3_ (2.18 eV). Figure 4a shows the PL mechanism of FAPbBr_3_ at 567 nm obtained under 800-nm two-photon absorption. Under 800-nm back-side illumination at a fixed bias of 5 V, the tendency of light current variation with respect to voltage (30–110 mW cm^−2^) is provided in Fig. 4b. Under ideal conditions, the photocurrent generated by two-photon absorption showed a square (*n* = 2) dependence on the input intensity [51]. However, our tested photocurrents (with 1 < *n* < 2) exhibited highly dependent on incident power, which was mainly attributed to the effect of trap-state’s sub-gap absorption [8, 52]. This tendency was consistent with that reported for near-infrared CsPbBr_3_ and MAPbBr_3_ photodetectors [8, 51]. Consequently, the responsivity of the two-photon pumped FAPbBr_3_ MC detector increased with increasing input intensity in the linear excitation region (Fig. 4c). The largest responsivity under 800-nm excitation (0.07 A W^−1^) was much higher than that previously reported for MAPbBr_3_ single crystals [51]. The fast response of our detector under two-photon excitation with a fast fall time (0.72 ms) is also shown in Fig. 4d. The result is similar to that observed under one-photon excitation (0.75 ms). The rapid pulse light with periodic changes was responsible for the missing rising stage.

Moreover, the amplified spontaneous emission (ASE) behavior of the FAPbBr_3_ microcrystalline film was measured using 800-nm laser pulses with tunable intensities. The emission spectra (Fig. S6) with increasing light intensity exhibited an ASE threshold of 1.75 mJ cm^−2^, which was similar to the reported two-photon ASE threshold of MAPbBr_3_ photodetectors (2.2 mJ cm^−2^) [53].

## Conclusion

In summary, organolead trihalide perovskite FAPbBr_3_ MCs were synthesized and then deposited as a photodetector. The photodetector exhibited a good responsivity of up to 4000 A W^−1^ under back-side one-photon excitation with an EQE and detectivity of up to 1.05 × 10^6^% and 3.87 × 10^14^ Jones, respectively. Besides, the two-photon responsivity under 800-nm excitation was 0.07 A W^−1^, which was four orders of magnitude higher than that reported for MAPbBr_3_ single crystals (10^−6^ A W^−1^). This deposited FAPbBr_3_ microcrystalline photodetector showed great potential for developing fast and sensitive photodetectors.

## Electronic supplementary material

Below is the link to the electronic supplementary material.

Supplementary material 1 (DOC 9234 kb)
