# Supplementary material for: Formamidinium Lead Bromide (FAPbBr3) Perovskite Microcrystals for Sensitive and Fast Photodetectors
Source: Nanomicro Lett. 2018 Apr 2;10(3):43. doi: 10.1007/s40820-018-0196-2 (PMC6199088; doi:10.1007/s40820-018-0196-2)
Supplement: Supplementary file 1 — Supplementary material 1 (DOC 9234 kb) [file 40820_2018_196_MOESM1_ESM.doc]

Supporting Information for

**Formamidinium Lead Bromide (FAPbBr_3_) Perovskite Microcrystals for Sensitive and Fast Photodetectors**

Fengying Zhang^1, 2^, Bin Yang^2^, Kaibo Zheng^3^, Songqiu Yang^2^, Yajuan Li^2^, Weiqiao Deng^2^, *, Rongxing He^1^, *

^1^Key Laboratory of Luminescence and Real-Time Analytical Chemistry (Southwest University), Ministry of Education, College of Chemistry and Chemical Engineering, Southwest University, Chongqing 400715, People’s Republic of China

^2^State Key Laboratory of Molecular Reaction Dynamics, Dalian Institute of Chemical Physics, Chinese Academy of Science, Dalian 116023, People’s Republic of China

^3^Department of Chemical Physics and NanoLund Chemical Center, Lund University P.O. Box 124, 22100 Lund, Sweden

*Corresponding authors. E-mail: [dengwq@dicp.ac.cn](mailto:dengwq@dicp.ac.cn) (Weiqiao Deng); [herx@swu.edu.cn](mailto:herx@swu.edu.cn) (Rongxing He)

**Supplementary Figures**


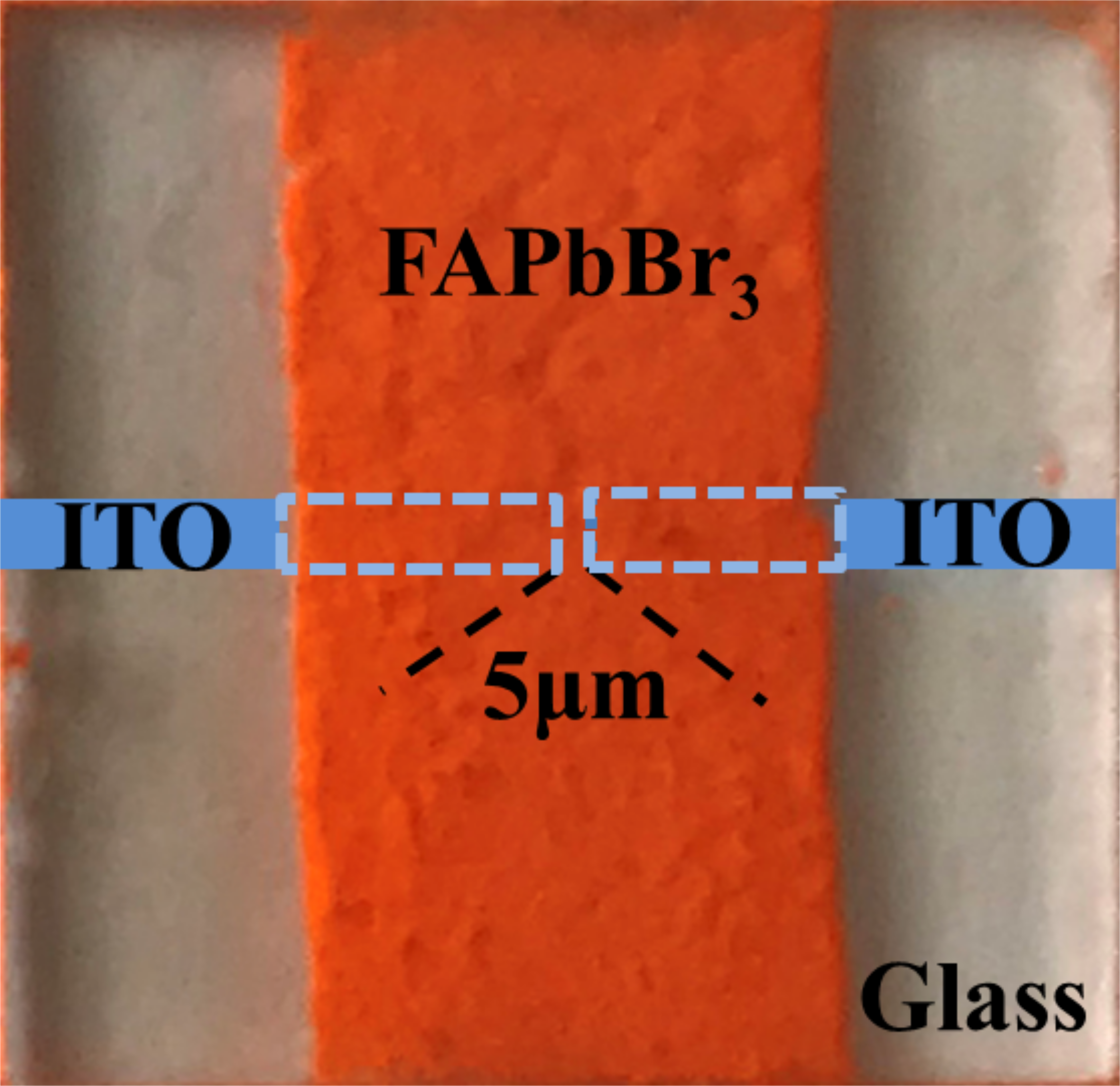


**Fig. S1** Schematic diagram of FAPbBr_3_ microcrystalline deposited photodetector


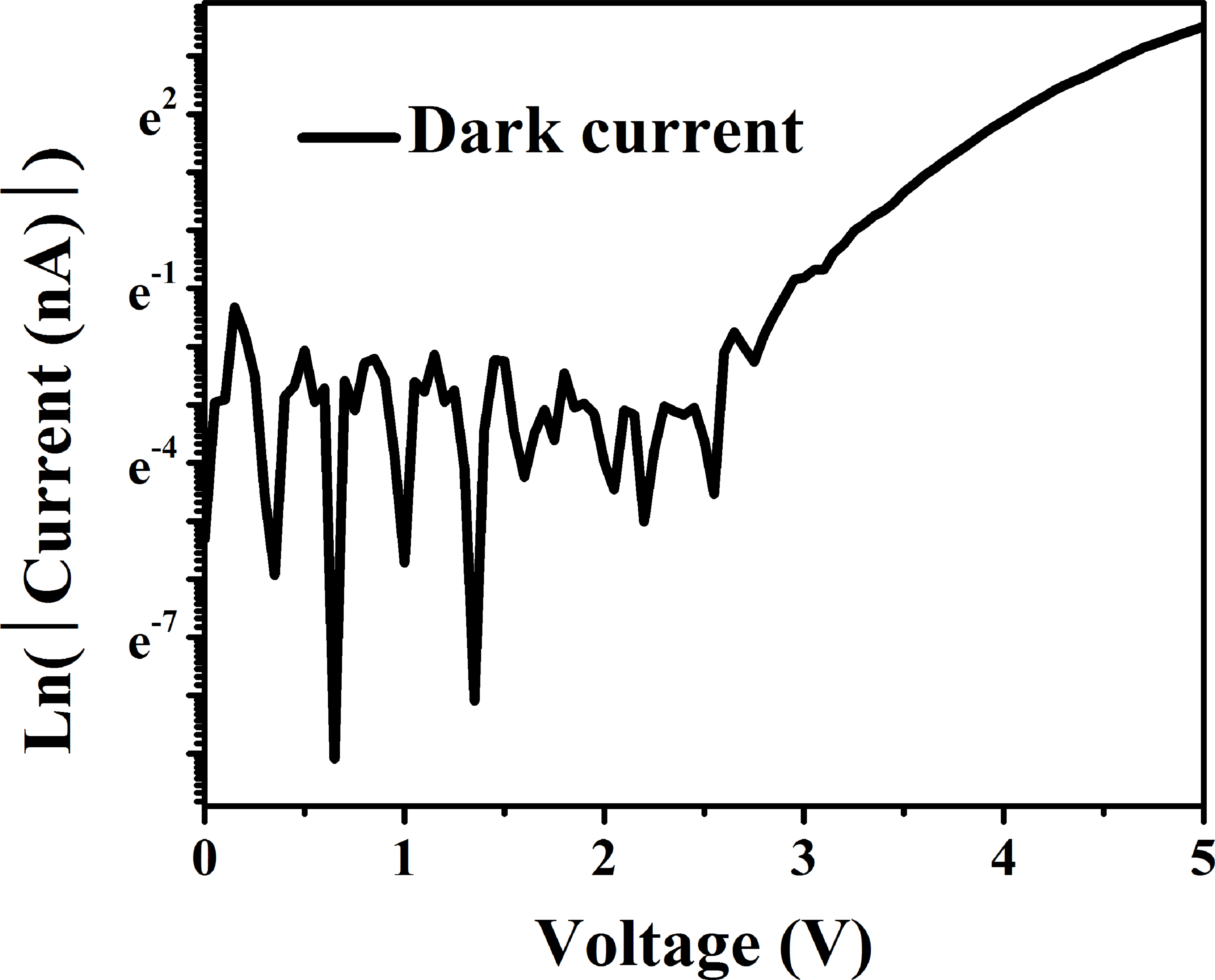


**Fig. S2** Current-voltage characteristic of FAPbBr_3_ microcrystalline photodetector in the dark


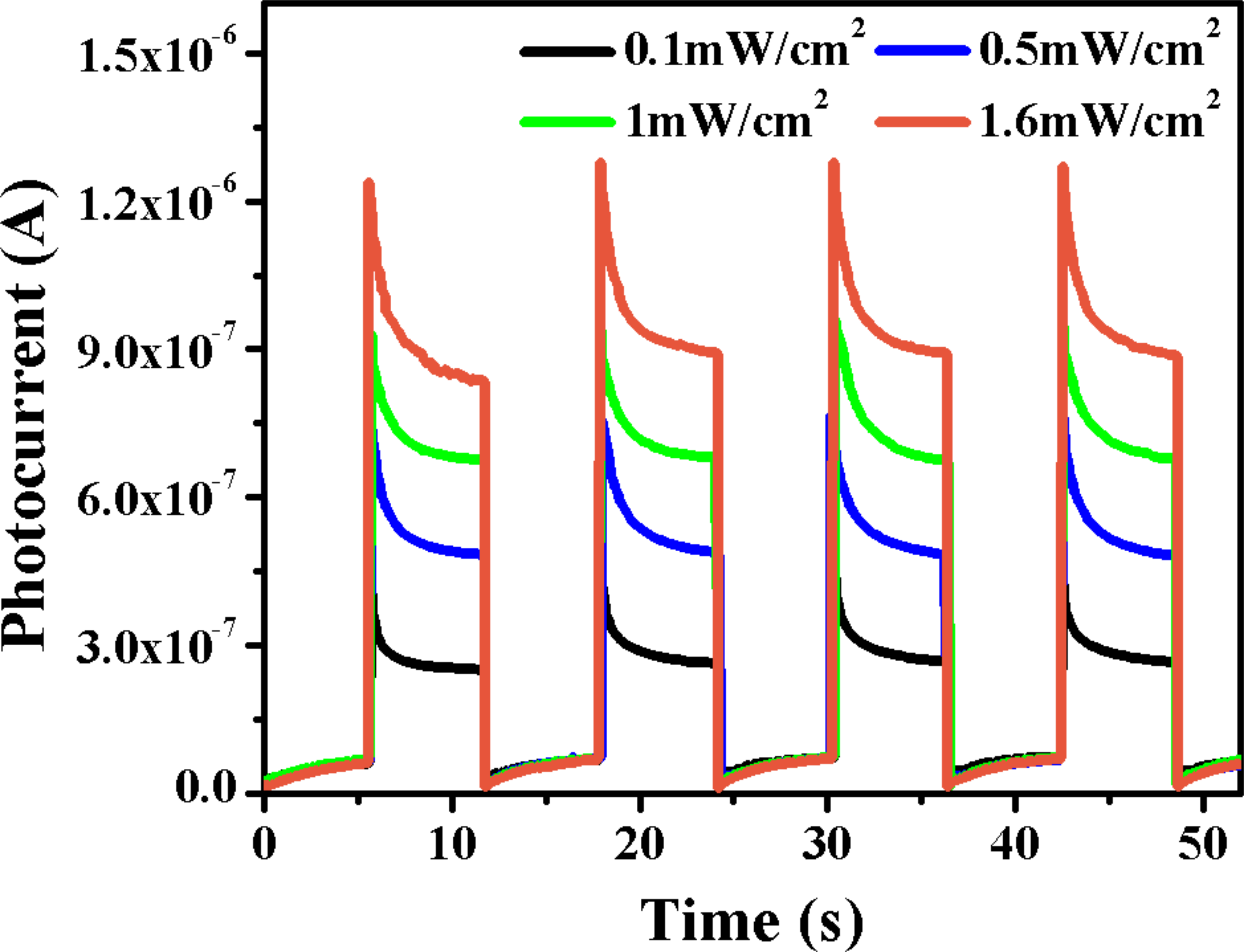


**Fig. S3** Transient photocurrent of the photodetector measured at bias voltages of 5V with changed incident light power of 495 nm


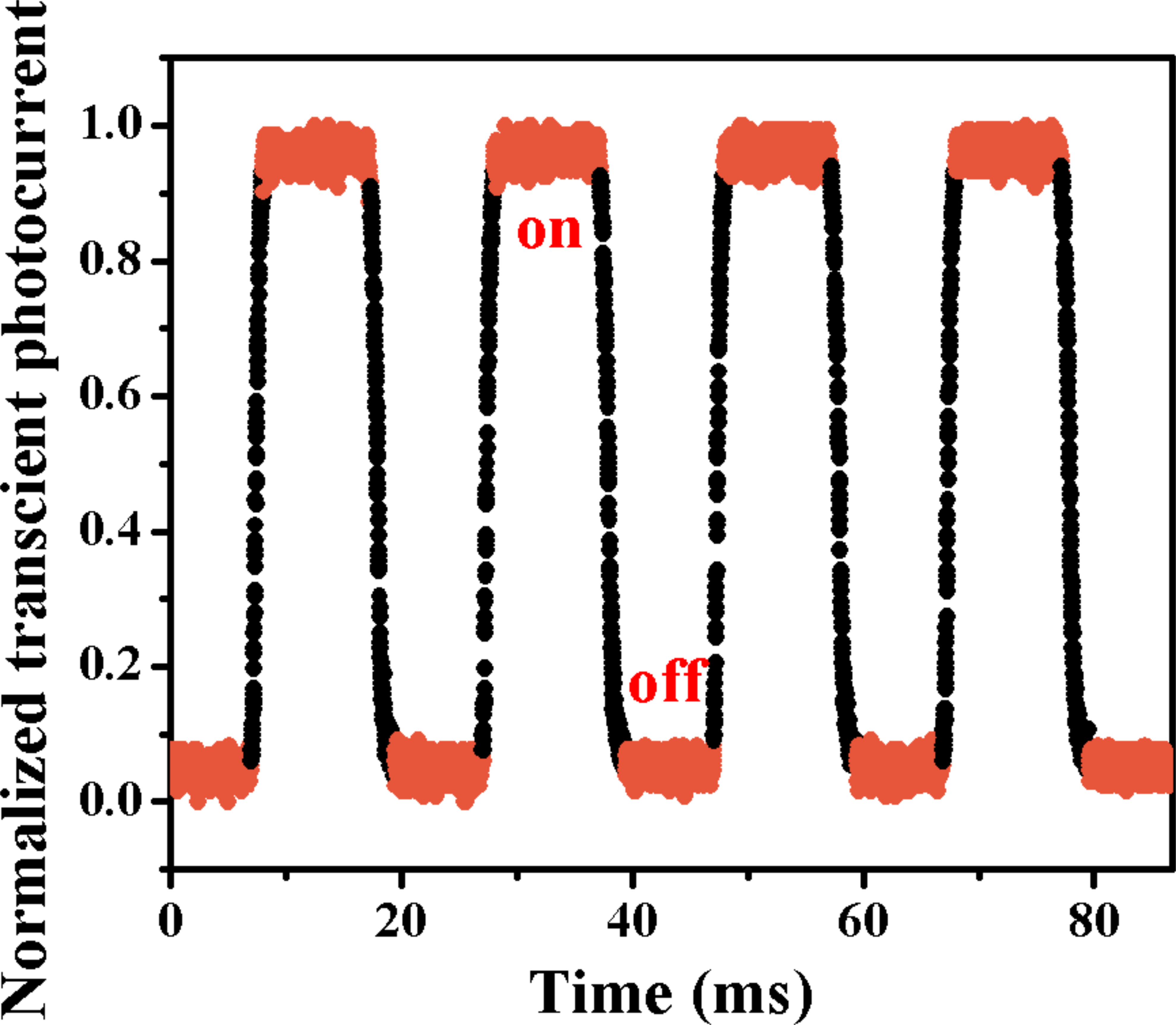


**Fig. S4** Response time of FAPbBr_3_ MCs photodetector with periodic irradiation of 400 nm monochromatic light


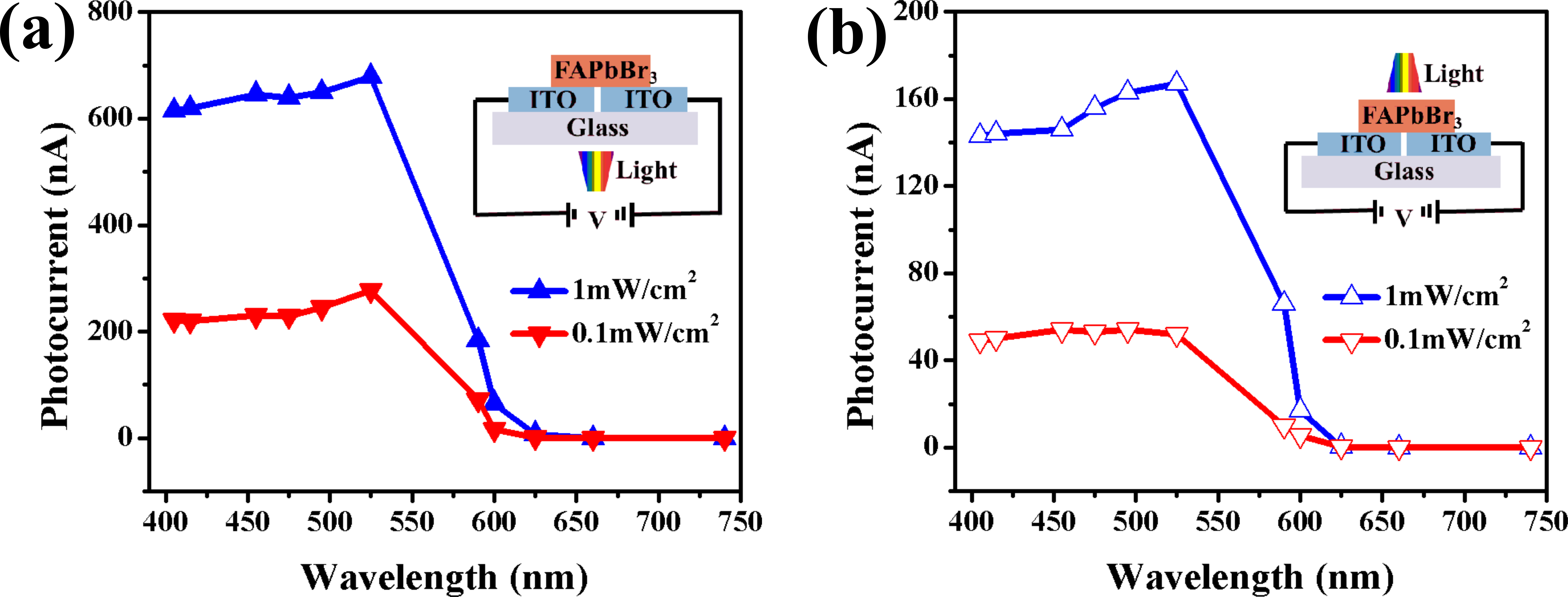


**Fig. S5** Photocurrent of FAPbBr_3_ photodetector under different incident light power (1 and 0.1 mW cm^-2^) upon **a** bottom illumination and **b** top illumination


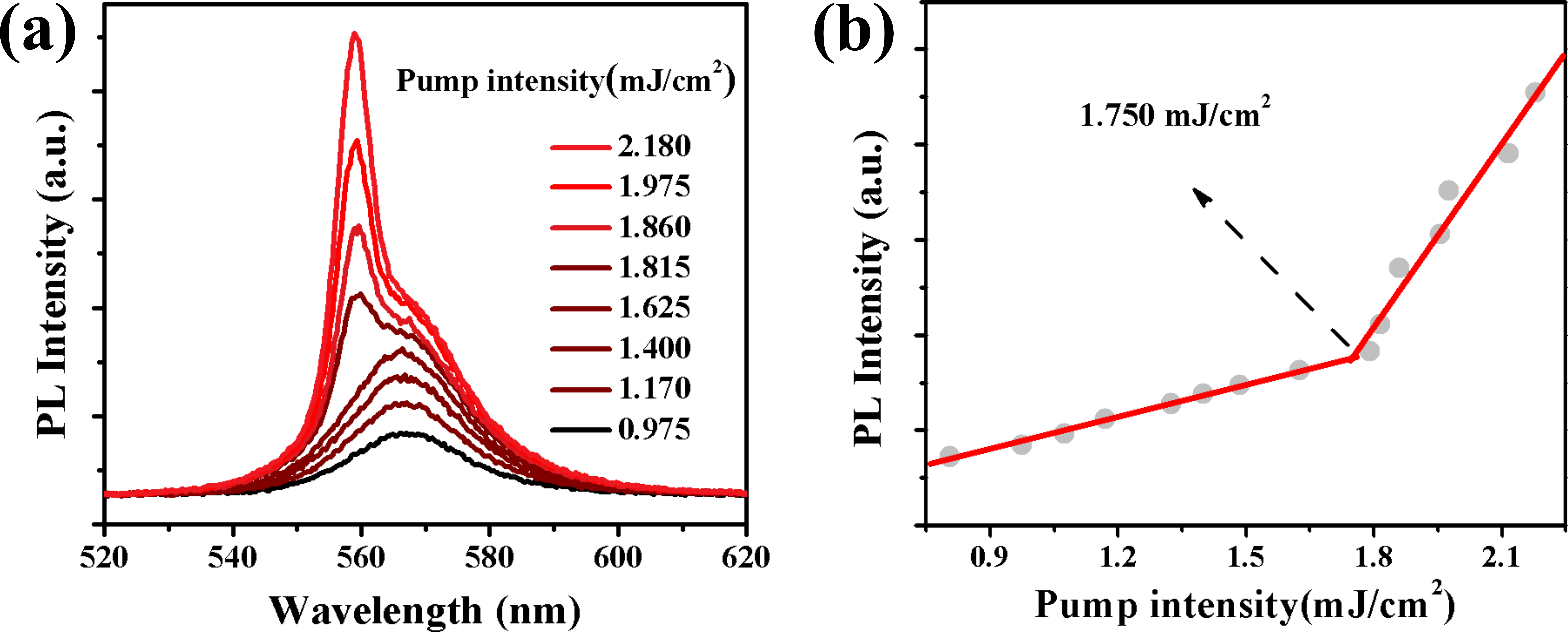


Fig. S6 a Pump intensity-dependent PL spectra and b integrated PL intensity versus pump intensity for FAPbBr_3_ MCs film under the irradiation of 800 nm
